# Supplementary material for: One dimensional wormhole corrosion in metals
Source: Nat Commun. 2023 Feb 22;14:988. doi: 10.1038/s41467-023-36588-9 (PMC9946947; doi:10.1038/s41467-023-36588-9)
Supplement: Supplementary file 1 — Supplementary information [file 41467_2023_36588_MOESM1_ESM.pdf]

Supplementary Materials for

One Dimensional Wormhole Corrosion in Metals

Authors: Yang Yang[1,2,†,*], Weiyue Zhou[3,†], Sheng Yin[4], Sarah Y. Wang[5], Qin Yu[4], Matthew J. Olszta[6], Ya-Qian Zhang[5], Steven E. Zeltmann[5], Mingda Li[3], Miaomiao Jin[7], Daniel K. Schreiber[6], Jim Ciston[1], M. C. Scott[1,5], John R. Scully[8], Robert O. Ritchie[4,5], Mark Asta[4,5], Ju Li[3,9], Michael P. Short[3,*], Andrew M. Minor[1,4,5,*]

Affiliations:

[1] National Center for Electron Microscopy, Molecular Foundry, Lawrence Berkeley National Laboratory, Berkeley, CA, USA.

[2] Department of Engineering Science and Mechanics and Materials Research Institute, The Pennsylvania State University, University Park, PA, USA

[3] Department of Nuclear Science and Engineering, Massachusetts Institute of Technology, Cambridge, MA, USA

[4] Materials Sciences Division, Lawrence Berkeley National Laboratory, Berkeley, CA, USA.

[5] Department of Materials Science and Engineering, University of California, Berkeley, CA, USA. MA, USA.

[6] Energy and Environment Directorate, Pacific Northwest National Laboratory, Richland, WA, USA.

[7] Department of Nuclear Engineering, The Pennsylvania State University, University Park, PA, USA

[8] Department of Materials Science and Engineering, University of Virginia, Charlottesville, VA, USA

[9] Department of Materials Science and Engineering, Massachusetts Institute of Technology, Cambridge, MA, USA.

[†] These authors contributed equally

* Email: yang@psu.edu (Y.Y.), hereiam@mit.edu (M.P.S.), aminor@berkeley.edu (A.M.M.)

This file includes:

Supplementary Notes 1 to 3
Supplementary Discussion
Supplementary Figures. 1 to 10
Supplementary Reference

Supplementary Note 1. The effect of grain boundary migration on the salt penetration speed

It is important to discuss the effect of grain boundary migration on the salt penetration speed. The understanding of this question could potentially inspire methods to slow the penetration of salt in metals.

Grain boundary migration can have two effects on the salt penetration:

- (1) The curved GBs elongate the total length of salt infiltration pathways, potentially slowing down corrosion. If the penetration speed v, defined as the distance along the grain boundary (GB) that salt etches per second, is a constant, then this effect will slow the penetration.
- (2) The diffusion-induced grain boundary migration (DIGM) process will gradually change local GB inclinations. If the penetration speed v is inclination-dependent, then DIGM will better focus the salt front to find a faster etching route along GBs. This in turn will facilitate salt penetration. Here, the penetration speed v is no longer a constant. Instead, it is dependent on the local GB misorientation and inclination.

A 2D Monte Carlo simulation (see Materials and Methods section) is provided to visualize the above two effects. The results are shown in Supplementary Figure 2 and Supplementary Movie 4. We compare the salt penetration in three different cases: (i) without DIGM and $v = 1$; (ii) with DIGM and $v = 1$; (iii) With DIGM and $v = 1 + 2 \cdot \tan|\theta|$, where θ is the GB inclination angle (the angle between the tangent line of GB and the horizontal axis, as illustrated in Supplementary Figure 2d). This equation is chosen such that v is higher when the inclination is closer to $\pm 90°$. There is not any specific physical model underlying this equation, but the angular dependence is included to demonstrate qualitatively the effect that changes in inclination can have on the penetration rate, if those inclinations lead to higher transport along the grain boundary.

From Supplementary Figure 2 and Supplementary Movie 4, it is shown that the penetration efficiency of (ii) is significantly lower than (i), while (iii) is the fastest case. This simulation result agrees with our experiments in which we do observe fast penetration of salt in metal. Therefore, we believe that the effect (2) is more significant than (1), and thus DIGM has an overall deleterious effect on the corrosion process. If the GB migration can be mitigated or stopped by means of GB engineering, one may be able to slow the salt penetration.

Supplementary Note 2. Vacancy mapping

Note: A small portion of the discussion from the paper is reproduced here for continuity of the discussion. The portion from the paper is expanded upon in this Supplementary Note.

This decrease in the lattice spacing within the DIGM zones can be interpreted as a type of phase transformation induced by the changes in Cr and vacancy fractions while the crystallographic symmetry is maintained. In this vein, the whole dealloying process can be pictured in a phase map that can be calculated by DFT simulations. This phase map, as shown in Fig. 4b, describes the intrinsic relationship between the relaxed lattice constant a_0, the Cr fraction C_{Cr}, and the vacancy fraction C_v. Starting from pristine Ni-20Cr (upper right corner of the triangle in Fig. 4b), the dealloying process shifts the position of the DIGM zone within the triangle map by altering C_{Cr} and C_v. The upper line of this triangle represents "100% replacement" meaning that for each Cr atom removed from the lattice, a Ni atom will replace it. Similarly, the bottom boundary of the triangle represents 0% replacement, indicating that no atom will refill the lattice site when a vacancy is created. One should also note that the relaxed lattice constant a_0 can be related to the actual lattice spacing a and the elastic strain ε^e by:

$$\varepsilon^e = (a - a_0)/a_0 \quad , \tag{1}$$

where a and C_{Cr} can be measured by 4D-STEM and EDX, respectively. If either the elastic strain ε^e in the dealloyed zones or C_v can be obtained, then the other can be derived. For the non-DIGM zones (1ii and 2ii), we will assume that the vacancy fraction is the same as that before corrosion. Thus, we can deduce the elastic strain in these regions directly from analyzing Fig. 4d. The strain in 1ii and 2ii are shown in Supplementary Figure 3d. For the DIGM zone (1i and 2i), however, the accurate measurement of elastic strain is nontrivial as ε^e and C_v are coupled. Here, we apply the theory of Eshelby's inclusion[1] to estimate of the elastic strain inside the DIGM zones.

A schematic drawing in Supplementary Figure 4 shows Eshelby's thought experiment for an inclusion problem, which assumes that the dealloying process is a kind of phase transformation with negative eigenstrain. When we take the dealloyed zone out and allow it to relax, the reduced lattice parameter from the dealloying process causes the zone to shrink (Supplementary Figure 4a). Next, when we return this dealloyed zone to the matrix, the region around the interface inside the matrix will have to be under tensile stress in order to accommodate the deformation in the dealloyed zone. Such tensile stress is indeed captured and verified by our strain mapping results shown in Supplementary Figure 3d. According to Eshelby's inclusion theory, we assume the dealloying process will lead to an eigenstrain of $\boldsymbol{\varepsilon}^*$ in the dealloyed region, and the corresponding elastic strain is equal to $\boldsymbol{\varepsilon}^e = \boldsymbol{S}\boldsymbol{\varepsilon}^* - \boldsymbol{\varepsilon}^*$, where $\boldsymbol{S}$ is the Eshelby tensor. Outside the dealloyed region, there is a strain jump $\boldsymbol{\Delta\varepsilon}$ between the inclusion and matrix at interface, which can also be expressed analytically for certain shapes of the inclusions [2–4]. From the analytical solutions of the Eshelby tensors for various shapes, we learned that the elastic strain in the inclusion ($\boldsymbol{\varepsilon}^e$) is of the same order of magnitude as the strain at the interface in the matrix ($\boldsymbol{S}\boldsymbol{\varepsilon}^* + \boldsymbol{\Delta\varepsilon}$). Therefore, based on the solution for a spherical Eshelby inclusion, we used the measured maximum strain near the interface in the matrix to calculate the $\boldsymbol{\varepsilon}^e$ in the inclusion, as an estimate of the elastic strain inside the DIGM zones. The estimated hydrostatic elastic strain for 1i and 2i in Fig. 4 is 0.178% and 0.277%, respectively. The averaged Cr fractions in 1i and 2i are 14.9 at. % and 13.9 at. %, respectively. The averaged lattice constant for 1i and 2i can then be calculated to be 0.3538 nm and 0.3533 nm under the estimated elastic strain, respectively. The averaged vacancy fractions in

1i and 2i are approximately 1.5×10^{-3} and 8.9×10^{-3}, respectively. The standard deviation of Cr fractions, lattice constant and vacancy fractions are plotted in Supplementary Figure 5 as error bars. These vacancy fractions are further compared with those in other systems in Fig. 4f. We found that the vacancy fractions in the DIGM zones are up to 10-100 times higher than those found in pure metals at the equilibrium melting point, and up to 1,000 to 10,000 times of that found in their equilibrium counterparts at 650°C.

Since C_v and ε^e are coupled in the DIGM zones, a precise measurement of elastic strain ε^e in these dealloyed regions would be difficult. However, using the two dashed lines in Fig. 4b to represent limits for the relaxed lattice constant, along with the measured lattice parameters, we can obtain the upper and lower limit of the strain maps for 1i and 2i, as shown in Supplementary Figure 3e and 3f. The elastic strain in these DIGM regions obtained in this manner is tensile and the local strain maximum ranges from 0.3% to 3%.

The major uncertainties of this vacancy calculation are from two parts:

(1) Accuracy of the elastic strain in the DIGM zone. We used the Eshlby's inclusion theory to estimate the strain field in the DIGM zone. We assumed that the DIGM had a uniform and small elastic strain field, because the material was heated at a high temperature (650˚C).

(2) Accuracy of the lattice parameters. We considered the average lattice spacing far away from the corrosion region and with uniform strain as the reference lattice spacing for the pristine sample. This approximation will lead to uncertainty due to the influence of the defects such as dislocations. However, the error resulted from this term is considered small as averaging was performed over a large and relatively uniform volume.

Supplementary Note 3. Mechanism of DIGM in the molten salt environment

The excess vacancy fraction found in the DIGM zones is proposed to be the precursor of intergranular voids during corrosion in a molten salt environment. Therefore, we present the atomic mechanism of DIGM in molten salt by modifying the previous DIGM theory by Broeder [5]. The atomic process of DIGM is schematically illustrated in Supplementary Figure 6.

I. Cr and Ni atoms from grain 1 and 2 will "jump" into the GB because the GB contains free volumes (vacancies) (Supplementary Figure 6a).
II. Cr at the GB leaches out and rapidly diffuses to the metal/salt interface, leaving vacancies behind in the lattice (Supplementary Figure 6b).
III. The vacancies at the GB from the leached Cr will be filled by Cr and Ni atoms in grain 1 and grain 2, creating vacancies inside the nearest planes to the GB within grain 1 and 2 (Supplementary Figure 6c).
IV. As long as molten salt is present, Cr continues to leach out at GBs. Therefore, I – III continue progressing (Supplementary Figure 6d).
V. The vacancies accumulate along the GB, causing it to broaden. However, this broadening will increase the *interfacial* energy. After a certain threshold, the broadening of the GB is no longer energetically favorable. Instead, the random atoms along the GB will self-organize and deposit on one side of GB (*i.e.,* on top of a specific grain). As a result, the GB has migrated by an atomic layer. As shown in Supplementary Figure 6e, this additional layer is grown on the grain 2 epitaxially, yet it possesses a lower Cr fraction and contains excess vacancies.
VI. By repeating the process of I – V, the GB will continue migrating, and the dealloyed (DIGM) zone will grow increasingly thicker. A vacancy gradient is formed in the DIGM zone such that the atomic layer closer to the GB tends to have a higher vacancy fraction, as shown in Supplementary Figure 6f.
VII. The high concentration of vacancies in the top layer of the DIGM zone within grain 2 can easily form a void once it is in contact with the salt, leading to salt infiltration (Supplementary Figure 6g).
VIII. Once the void is formed, the atoms in grain 1 and 2 may still "jump" into the crevice, re-organize, and deposit on one of the grains. (Supplementary Figure 6h).
IX. Therefore, the crevice may meander like a river and change its location during the corrosion process (Supplementary Figure 6i).

Supplementary Discussion

Our naming of 1D does not specify ideal 1D, as characterized by a very high feature aspect ratio, neither "near atomic size" nor the tunneling into aluminum or porous silicon[6–8]. We took the top-down approach by realizing that GBs are 2D defects, while 1D wormholes partially cover GBs in high aspect ratio pathways. As 2D defects, GBs require five parameters to define. We use similar language to describe wires, ropes, and cables with diameters across scales. Therefore, we think that 1D does not necessarily need to be near-atomic size, as long as the diameters do not vary significantly along the path, and the aspect ratio is quite high. We believe our features exhibit this far higher aspect ratio, as opposed to corrosion pits which have been deemed "1D" in some prior studies, yet have a small aspect ratio of depth to diameter. Finally, "1D wormhole" together refers to the observed microstructure.

Here, we would like to illustrate the difference between our work and previous corrosion studies that directly/indirectly mentioned the "1D" concept. We show that the "1D wormhole corrosion" we reported in our manuscript is significantly different from previous studies.

1. Comparison between our work and previous work that mentioned "1D" and is indeed 1D according to our definition in the manuscript.

Previous observations of 1D rod-like microstructures, for example, in the etching of aluminum, silicon, titanium with ***strong acids***[6–8], are not directly from selective dissolution. In most cases, a single element system is used, and the microstructure is created by fast interfacial reactions without the participation of solid-phase diffusion or the pre-distribution of elements in an alloy. More importantly, the so-called "1D" holes in previous studies do not form a percolating network, and it does not involve the discussion of GBs.

In terms of corrosion of alloys in molten salt, our observation and confirmation of the 1D wormholes laying on the 2D grain boundaries is the first recorded instance that we could find. The shape of wormholes is not as ideal (or simple) as the 1D rod-like microstructures mentioned above, as previous studies consisted of delicate experiments to intentionally create the 1D rod-like microstructure. Our wormholes appear on their own, and are robust to changes in experimental conditions, resulting in a more generalizable phenomenon. We believe that the 1D wormholes are common microstructures in molten salt corrosion (or in high-temperature liquid lead corrosion), which are previously not correctly recognized as continuous or *percolating* structures. Currently, the most popular interpretation of these holes in the molten salt corrosion field is that these "voids" on the cross-sections are "Kirkendall voids[9]," which implies that the voids are discontinuous initially or partially discontinuous. For the corrosion of structural materials in high-temperature molten salts, we believe recognizing the 1D wormholes is important as it can

be a critical stage before 2D intergranular corrosion or other types of transgranular corrosion that initiate from GBs.

In short, we believe that 1D wormhole corrosion is a new dimensionality (1D + percolating pipelines along GBs) of corrosion in structural alloys, which must be minimized in engineering systems such as next generation nuclear power plants or concentrated solar power plants. By contrast, previous observations of 1D rod-like microstructures show a very different and non-percolating (noncapillary) morphology, and involve the use of strong acids and/or a non-structural material to create microstructures, which is more relevant in functional materials applications rather than structural materials applications.

2. Comparison between our work and previous work that mentioned "1D" and is in fact 2D according to our definition in the manuscript.

Ghaznavi et al. recently shown that "1D corrosion" can occur in $Fe_{52}Ni_{48}$ alloy after corrosion in a molten KCl mixture at 600˚C [10]. The "1D" corrosion they observed is a kind of "2D" corrosion based on the definition used in our manuscript. For the 1D wormhole corrosion in our paper, the void will be discontinuous (just like isolated dots) along GBs in a cross-section, as shown in Fig. 1 in this paper. In the paper by Ghaznavi et al., the voids look like long and continuous lines in the cross-sectional view, which is significantly different from our work. We assume that the 3D morphology of this slot-like voids in Ghaznavi et al.'s paper will look like platelet rather than 1D tunnels. Thus, according to our definition, the corrosion morphology in their paper may be a kind of "2D" corrosion. In addition, the 1D wormholes are along GBs, while those shown in Ghaznavi et al.'s paper are mostly intragranular voids.

3. Comparison between our work and previous work that mentioned "1D" and is in fact 3D according to our definition in the manuscript.

Previously, Pickering and Swann *et al.* mentioned "1D" in their dealloying papers[11,12]. We found the corrosion morphology they found is "3D"-like rather than "1D". The morphology of corrosion they referred to is the well-known morphology of dealloying, which is 3D based on our definition (See Fig.1 in the manuscript).

For Pickering's dealloying experiments, it was mainly ambient temperature aqueous dealloying. They explained the phenomenon by **volume diffusion** (originally by single vacancies, then changed to by divacancies). Later, researchers such as J. Erlebacher and K. Sieradzki have provided strong evidence that ambient temperature aqueous dealloying is via a percolation dealloying mechanism (with significant contribution from **surface**

diffusion) with a resulting bicontinuous structure[13]. The electron microscopy images from Pickering's papers seem to show bicontinuous structures. Also, the experimental conditions described in the papers agree with the conditions of dealloying experiments showing a bicontinous structure. In addition, their experiments of vaporization of Cu86Zn and Cu65Zn at elevated temperature produced either Kirkendall voids or a bicontinuous microstructure[14], the latter of which is also called vapor phase dealloying[15]. While the phase transformation of CuZn alloys during the dealloying makes the interpretation of the results challenging, none of Pickering's experiments is similar to our 1D wormholes. In their research of dealloying of Co-Pt alloys at elevated temperature in HCl vapor[16], the fraction of the less-noble element (Co) is between 70-90 at%, but the microstructure in their work looks like a bicontinuous structure or an extreme case of liquid metal dealloying even when the less noble element concentration is significantly high[17].

For Peter Swann's work on "corrosion tunneling," similar to Pickering's experiments, the Cu_3Au and $Cu_{3.5}Au$ are dealloyed at ambient temperature in ferric chloride solutions. While they call them "tunnels," the microstructure from their experiments is actually bicontinuous from a dealloying point of view. The tunnel seems to be the early stage of the formation of a bicontinuous structure. Although these corrosion tunnels may seem to suitably describe our 1D wormholes, the corrosion tunnels in Swann's work and our work show significantly different features. First, the concept of tunnel by Swann et al. is further explored by A. J. Forty and G. Rowlands, who theoretically predicted the tunneling results when the alloy has a low concentration of noble metal[18] ($c < ½$). Now with the high-density percolation dealloying theory, we know that this ½ is close to the parting limit of ambient temperature dealloying. So, the corrosion tunneling they refer to is very likely to be the bicontinuous structure. As we mentioned in our previous discussion, bicontinuous structures are 3D, while wormholes in this paper are regarded as 1D. Second, the tunnel observed in Swann's work show no intermediate range of lattice parameters in a diffusion zone and no elastic strain field near the tunnel[12], while our 4D-STEM experiments clearly indicated remarkable heterogeneity of lattice parameters in the diffusion-induced migration zone (Fig. 4 in main text).

In addition, while Swann et al mentioned "one-dimensional" in several of their other papers, we found that these are not used to describe the morphology of holes. Instead, the word "one-dimensional" is used to describe their simplified theoretical 1D diffusion model in which they assumed that the diffusion is normal to the surface (1967)[19], or an island of infinite width (1980)[18].

Based on the discussions above, we think the 1D wormholes reported in our manuscript are new and significantly different from previous work. However, we also found that a part of the theory of the "corrosion tunnel" by Swann[12] may be used to explain some of the features found in

1D wormholes. Swann proposed that "Lateral dissolution ceases when the tunnel walls become covered with gold (the more noble element). Possibly corrosion occurs more rapidly at the hemispherical base of the tunnel because the ratio of surface area to volume dissolved is greater there and the surface is not as well covered by gold." [12] This seems to be helpful for explaining the "focusing" mechanism in our 1D wormhole, which exhibits a very high aspect ratio. However, further in-depth studies are required to validate this theory.

Supplementary Figures

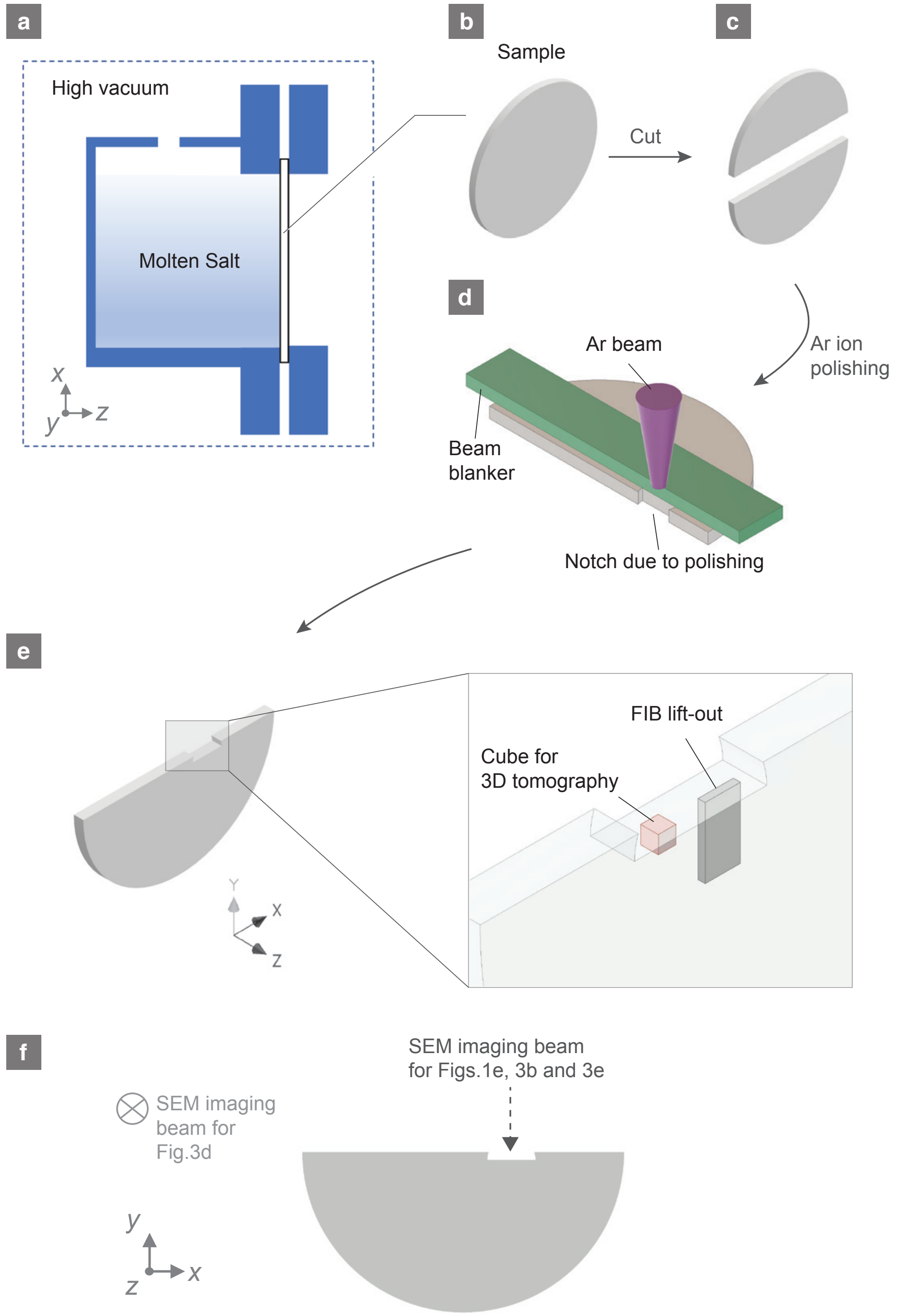

Supplementary Figure 1.

Illustration of the experimental setup, sample preparation, and imaging directions. a Schematic drawing of the molten salt corrosion cell, which is similar to a permeation test device. **b - e** Schematic drawing of the sample preparation process. **f** Schematic drawing illustrating the relation between SEM imaging direction and sample geometry.

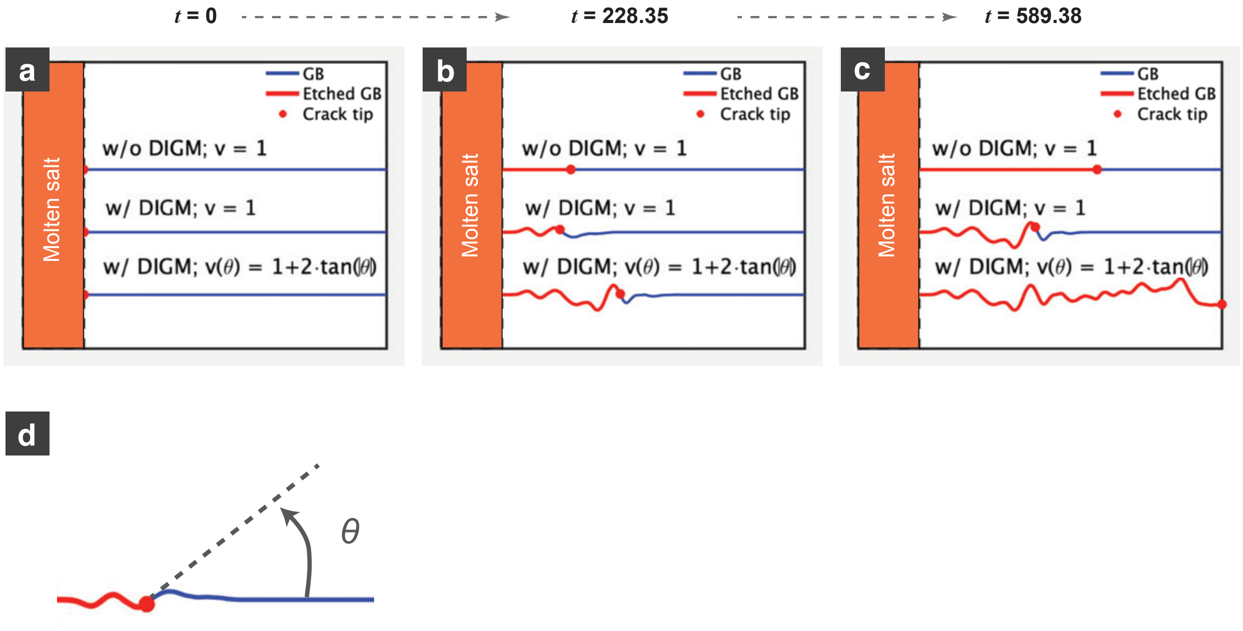

Supplementary Figure 2.

Monte Carlo simulation comparing the penetration speed of salt under three different conditions: (i) without DIGM and $v = 1$; (ii) with DIGM and $v = 1$; (iii) with DIGM and $v = 1 + 2 \cdot \tan|\theta|$. a - c correspond to $t = 0$, $t = 228.35$, and $t = 589.38$, respectively. **d** Definition of the GB local inclination angle θ.

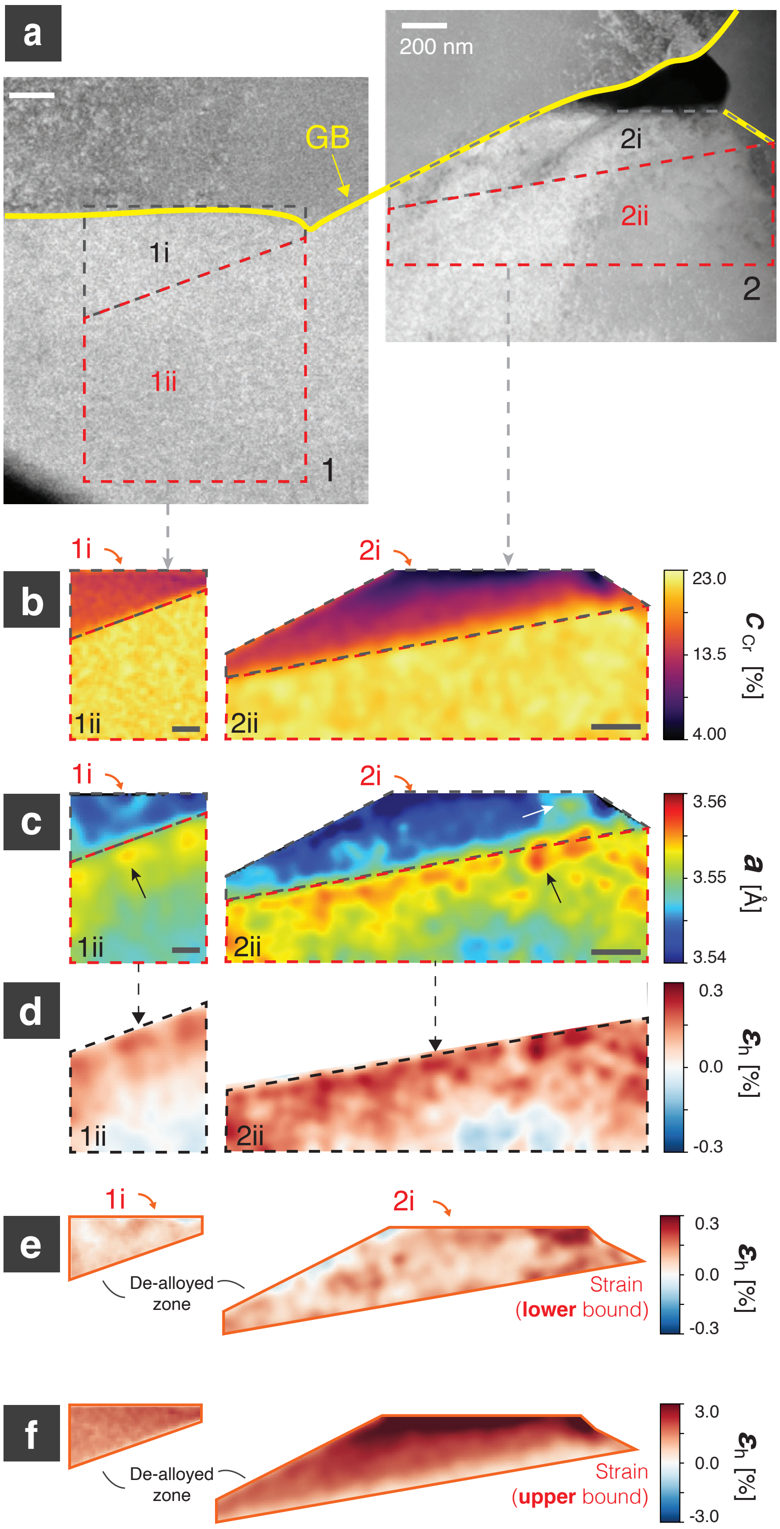

Supplementary Figure 3.

Additional data for the vacancy mapping method. a–c Same as Fig. 4. **d** Hydrostatic strain in the non-DIGM zones (1ii and 2ii). **e–f** Upper bound and lower bound of the hydrostatic strain in the DIGM zones (1i and 2i). All scale bars are 200 nm.

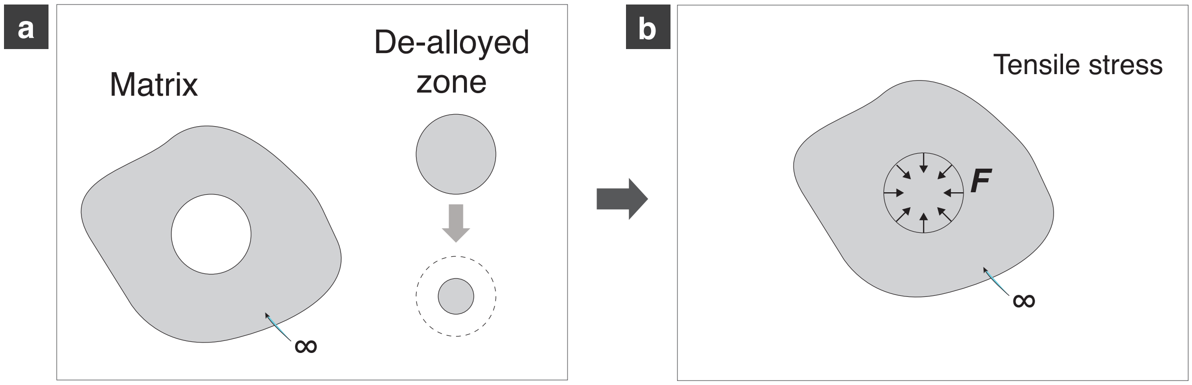

Supplementary Figure 4.

a-b Step-by-step schematic drawing of the Eshelby's inclusion diagram for the dealloying case assuming an infinitely large matrix.

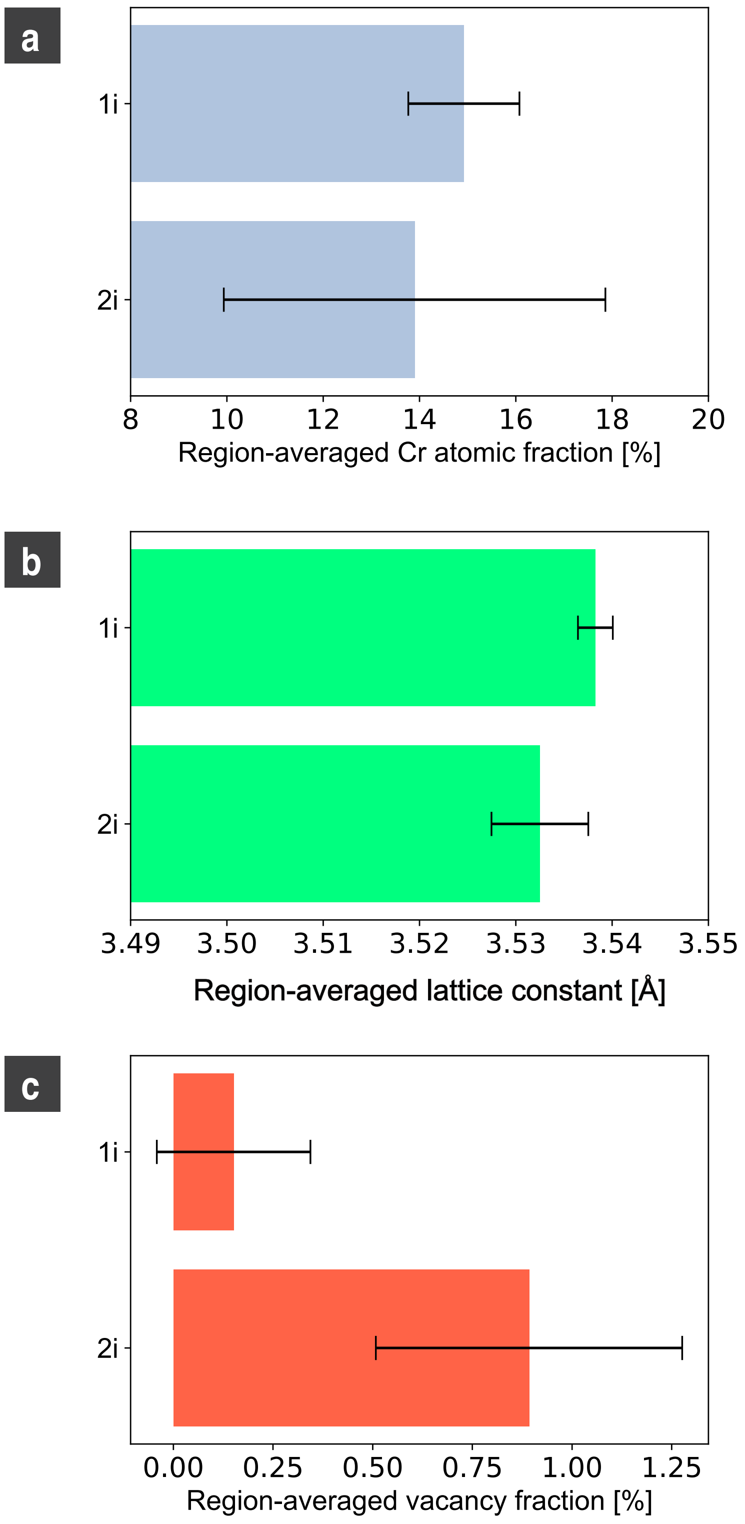

Supplementary Figure 5.

a-c Comparison of the averaged Cr fraction, averaged relaxed lattice constant, and averaged vacancy fraction in region 1i and 2i, respectively. The error bars show the standard deviation of the data.

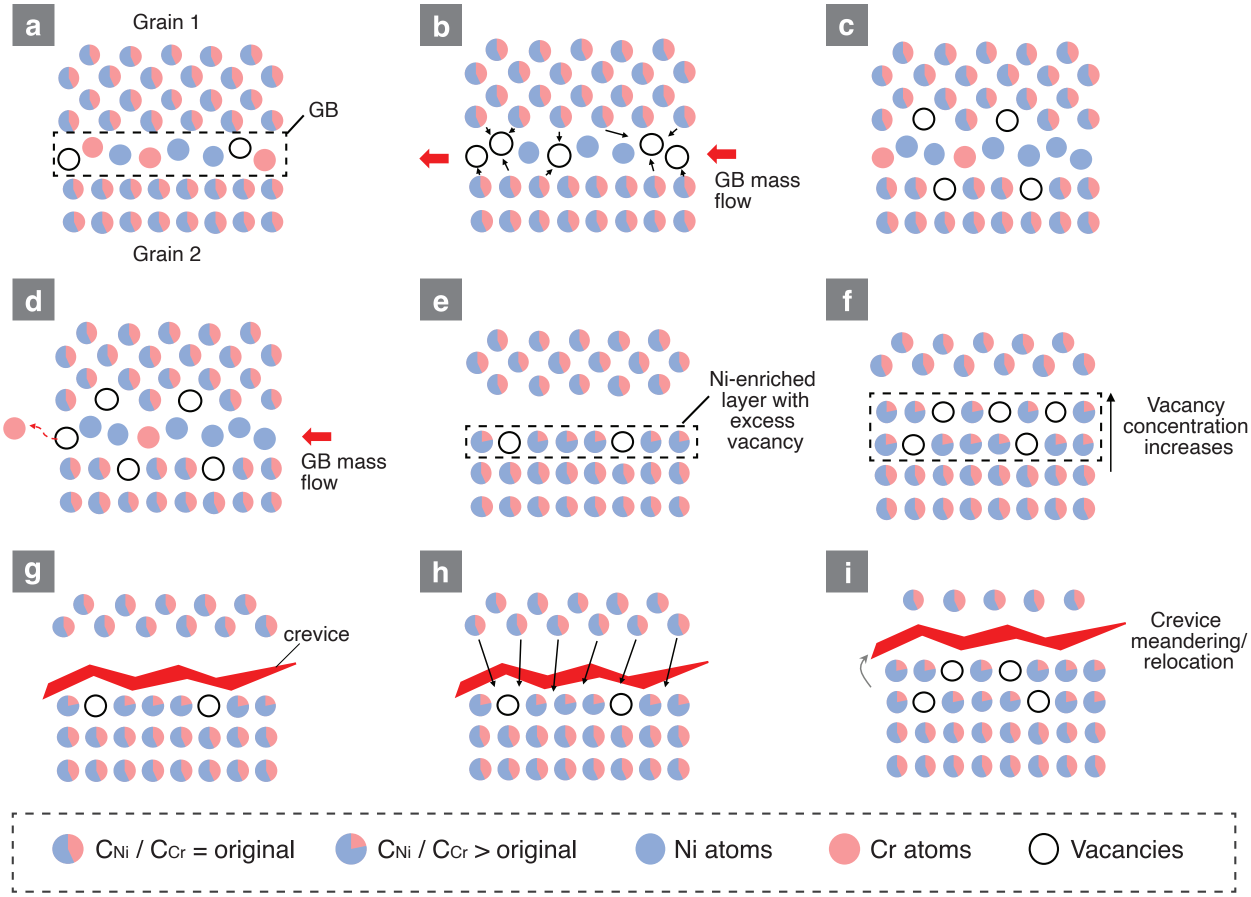

Supplementary Figure 6.

a-i Step-by-step schematic drawing of the DIGM process in molten salt with the consideration of vacancy supersaturation in the DIGM zone. The multicolored circles represent an atom-sized volume of sample with the same composition as the Ni-20Cr alloy.

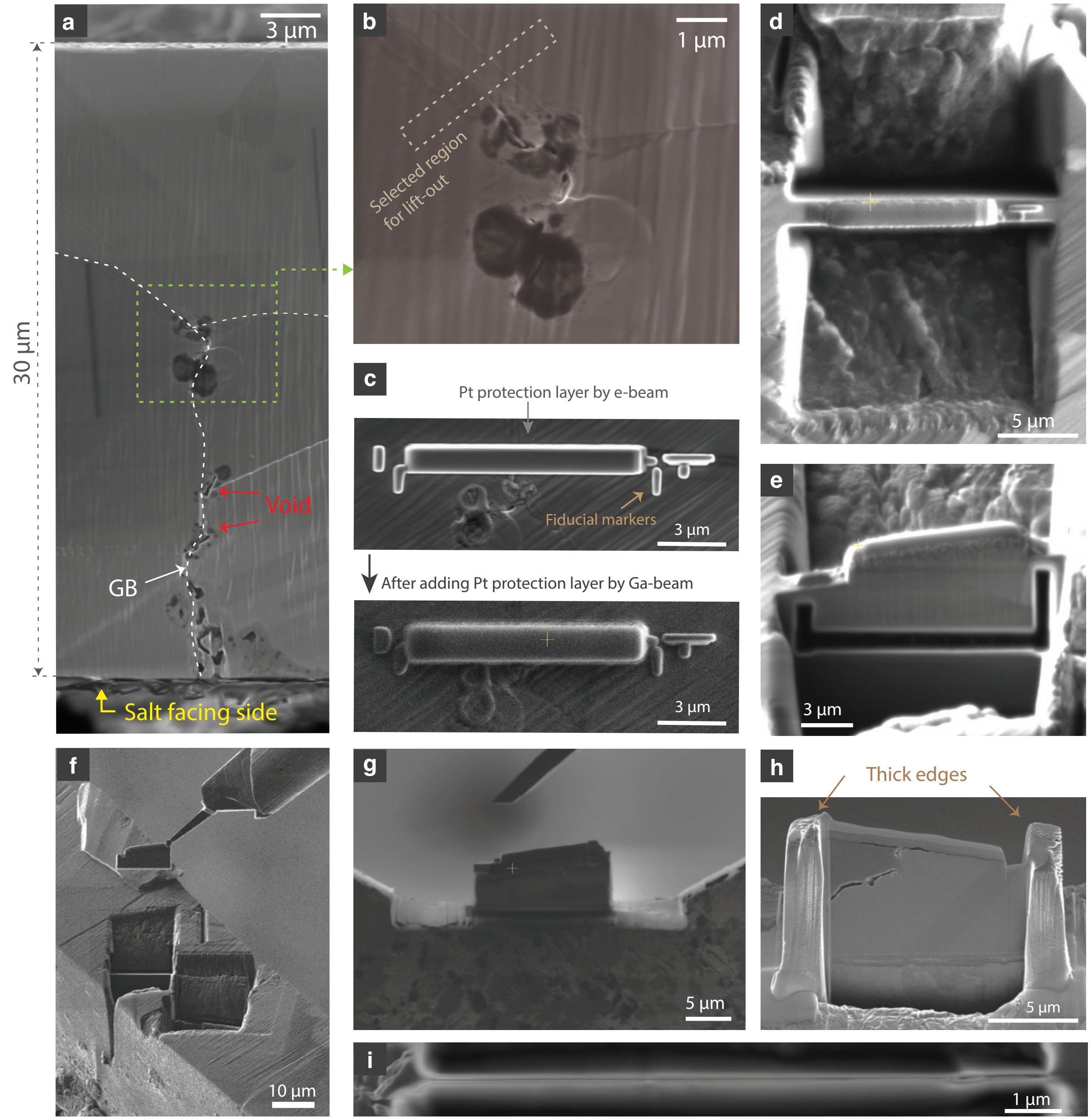

Supplementary Figure 7.

A typical FIB lift-out process in our experiment. a Overview of the sample before lift-out. We first identified the GBs and then select a region (boxed) in the middle that contains a GB. **b** An enlarged view of the boxed region in **a**. **c** Deposition of fiducial markers and Pt caps to protect the sample surface. **d** Milling the bulk trench. **e** U-cuts to free the lamella from the trench. **f** Lifting-out the lamella using the Easy-lift needle. **g** Attached the lamella on a V-notch of the FIB half-grid. **h** Thinning of the lamella from both sides. **i** SEM image showing the thickness of the lamella after thinning.

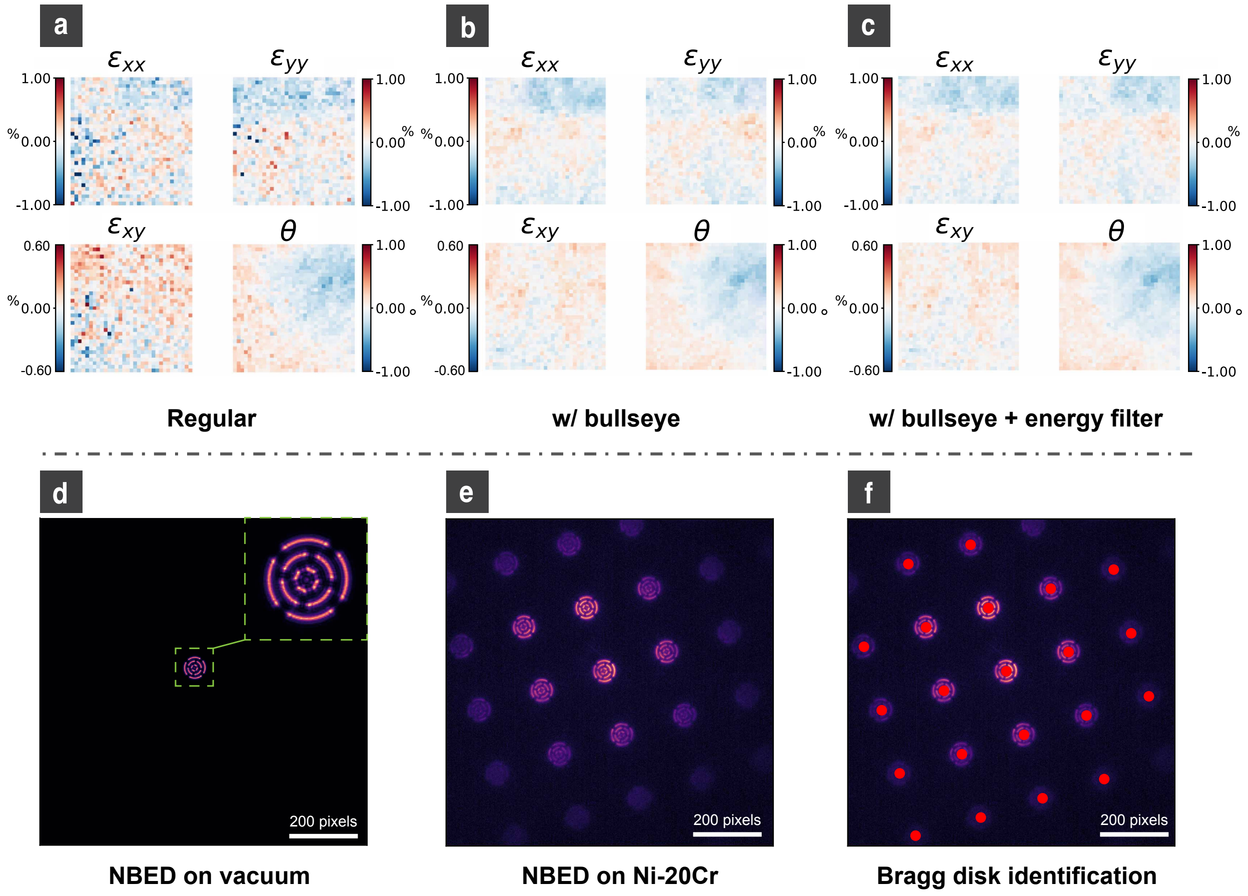

Supplementary Figure 8.

Evidence showing that the bullseye aperture and the energy filter is critical for enhancing the accuracy of lattice parameter mapping. A comparison of lattice parameter maps on the same region of the same sample by different 4D-STEM experimental setups is presented: **a** with a traditional circular aperture, **b** with a bullseye aperture, and **c** with a bullseye aperture and an energy filter. Note that **b** and **c** look very similar because the range of the color-bar is selected to cover the whole range of values on the map. However, the tiny differences between **b** and **c** are still important for the accuracy of lattice parameter mapping. ε_{xx}, ε_{yy}, and ε_{xy} are three key components in the 2D strain tensor, representing the strain in *xx*, *yy*, and *xy* (shear) direction, respectively. θ is the local lattice rotation. **d-f** Illustration of the lattice parameter mapping process by 4D-STEM using a bullseye aperture. **d** A NBED pattern on vacuum (*i.e.,* the electron beam does not go through any samples), which is used as a template for Bragg disk identification; **e** A NBED pattern on the Ni-20Cr sample, where all Bragg disks are in bullseye shape because of the bullseye aperture; **f** A typical Bragg disk detection result analyzed by the py4DSTEM package. The higher order Bragg disks are very dim in brightness, but their centers can still be accurately detected due to the use of the unique bullseye C2 aperture.

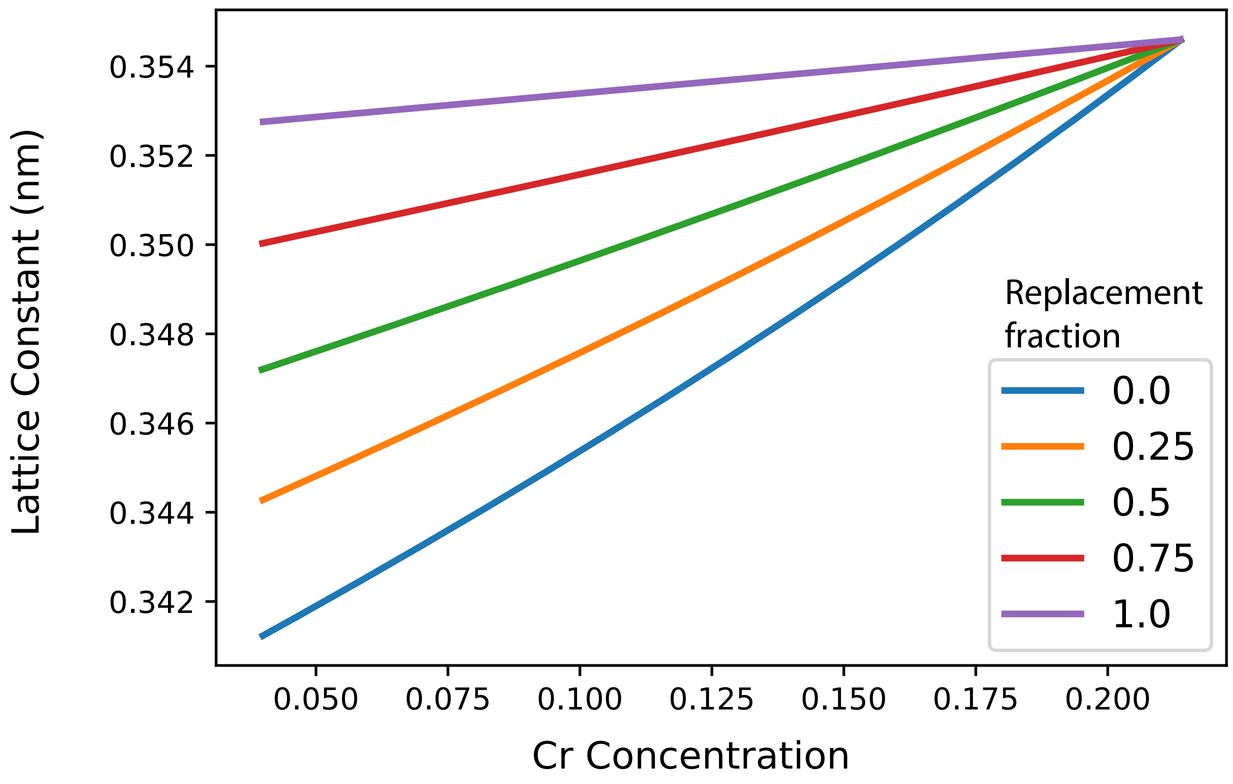

Supplementary Figure 9.

DFT modeling of the evolution of lattice constant during the dealloying process when the replacement fraction of depleted Cr with Ni is fixed at a constant. 0.0 replacement fraction corresponds to each depleted Cr becoming a vacancy site.

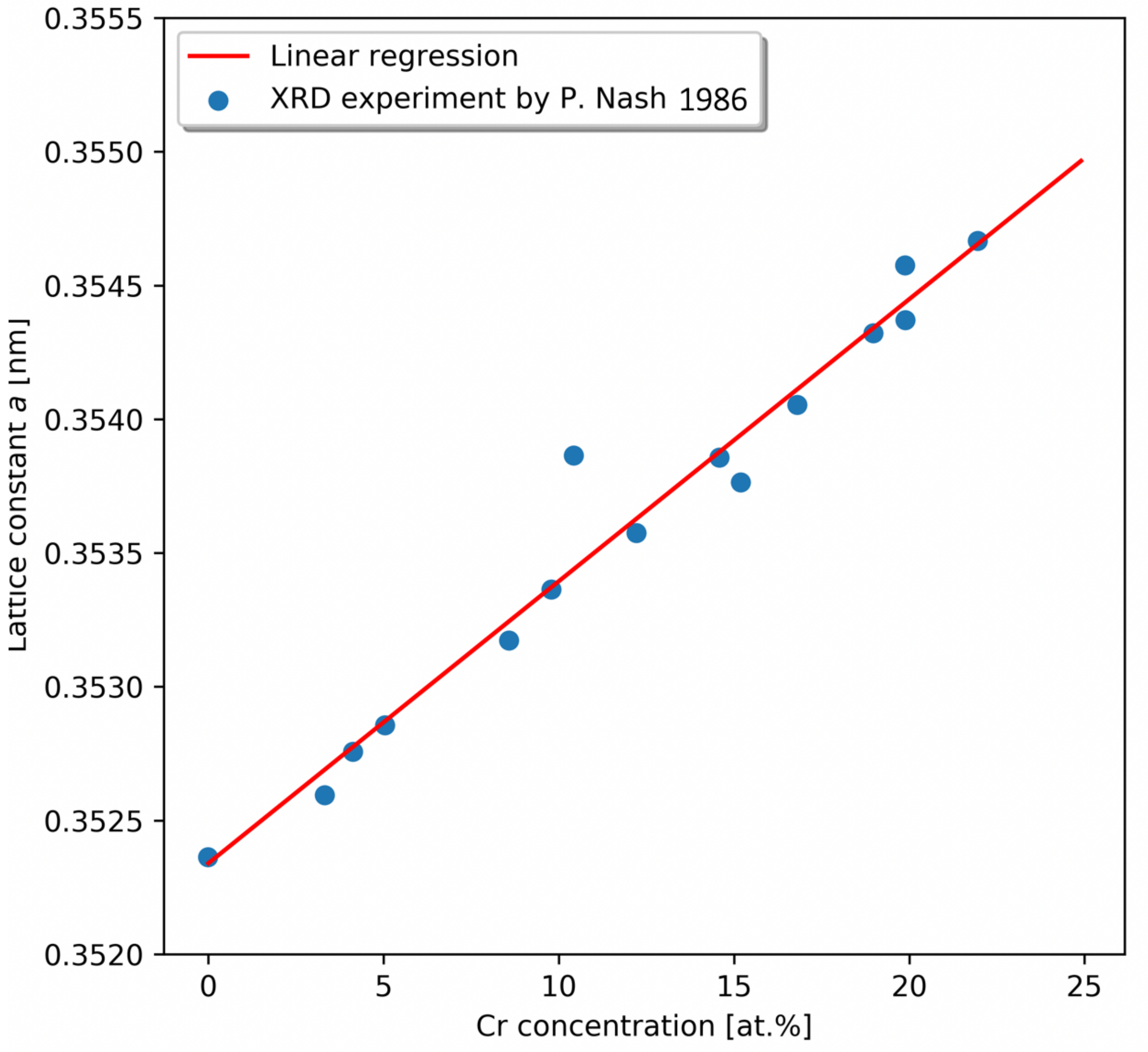

Supplementary Figure 10.

Experimental data from Ref[20] and linear fit showing the effect of Cr fraction on the lattice parameter in a Ni-Cr alloy when the vacancy fraction is negligible, corresponding to the 100% replacement case.

Supplementary References

1. The determination of the elastic field of an ellipsoidal inclusion, and related problems. *Proc. R. Soc. London. Ser. A. Math. Phys. Sci.* **241**, 376–396 (1957).
2. Jin, X., Keer, L. M. & Wang, Q. A closed-form solution for the Eshelby Tensor and the elastic field outside an Elliptic Cylindrical Inclusion. *J. Appl. Mech. Trans. ASME* **78**, (2011).
3. The elastic field outside an ellipsoidal inclusion. *Proc. R. Soc. London. Ser. A. Math. Phys. Sci.* **252**, 561–569 (1959).
4. Ju, J. M. & Sun, L. Z. A novel formulation for the exterior- point eshelby's tensor of an ellipsoidal inclusion. *J. Appl. Mech. Trans. ASME* **66**, 570–574 (1999).
5. Den Broeder, F. J. . Interface reaction and a special form of grain boundary diffusion in the Cr-W system. *Acta Metall.* **20**, 319–332 (1972).
6. Peng, N., Wen, Y., Shang, W. & He, Y. Formation and effect of the branched layer during the tunnel etching of aluminum foil. *J. Mater. Sci.* **55**, 1246–1255 (2020).
7. *Porous silicon: from formation to application: formation and properties*. (CRC Press, 2016).
8. Roy, P., Berger, S. & Schmuki, P. TiO2 nanotubes: Synthesis and applications. *Angew. Chemie - Int. Ed.* **50**, 2904–2939 (2011).
9. Ignatiev, V. & Surenkov, A. *Material performance in molten salts*. *Comprehensive Nuclear Materials* **5**, (Elsevier Inc., 2012).
10. Ghaznavi, T., Persaud, S. Y. & Newman, R. C. The Effect of Temperature on Dealloying Mechanisms in Molten Salt Corrosion. *J. Electrochem. Soc.* **169**, 111506 (2022).
11. PICKERING, H. W. & SWANN, P. R. Electron Metallography of Chemical Attack Upon Some Alloys Susceptible to Stress Corrosion Cracking. *Corrosion* **19**, 373t-389t (1963).
12. SWANN, P. R. Mechanism of Corrosion Tunnelling With Special Reference to Cu3Au. *Corrosion* **25**, 147–150 (1969).
13. Erlebacher, J., Aziz, M. J., Karma, A., Dimitrov, N. & Sieradzki, K. Evolution of nanoporosity in dealloying. *Nature* **410**, 450–453 (2001).
14. Pickering, H. W. Formation of New Phases during Anodic Dissolution of Zn-Rich Cu-Zn Alloys. *J. Electrochem. Soc.* **117**, 8 (1970).
15. Lu, Z. *et al.* Three-dimensional bicontinuous nanoporous materials by vapor phase dealloying. *Nat. Commun.* **9**, 1–7 (2018).
16. Pickering, H. W. & Kim, Y. S. De-alloying at elevated temperatures and at 298 k-similarities and differences. *Corros. Sci.* **22**, 621–635 (1982).
17. Geslin, P.-A., McCue, I., Gaskey, B., Erlebacher, J. & Karma, A. Topology-generating interfacial pattern formation during liquid metal dealloying. *Nat. Commun.* **6**, 8887 (2015).
18. Forty, A. J. & Rowlands, G. A possible model for corrosion pitting and tunneling in noble-metal alloys. *Philos. Mag. A Phys. Condens. Matter, Struct. Defects Mech. Prop.* **43**, 171–188 (1981).
19. Pickering, H. W. & Wagner, C. Electrolytic Dissolution of Binary Alloys Containing a Noble Metal. *J. Electrochem. Soc.* **114**, 698 (1967).
20. Nash, P. The Cr−Ni (Chromium-Nickel) system. *Bull. Alloy Phase Diagrams* **7**, 466–476 (1986).
