# Supplementary material for: One dimensional wormhole corrosion in metals
Source: Nat Commun. 2023 Feb 22;14:988. doi: 10.1038/s41467-023-36588-9 (PMC9946947; doi:10.1038/s41467-023-36588-9)
Supplement: Supplementary file 3 — Description of Additional Supplementary Files [file 41467_2023_36588_MOESM3_ESM.pdf]

Description of Additional Supplementary Files

File Name: Supplementary Movie 1
Description: FIB-SEM 3D reconstruction of a representative Ni-20Cr sample after corrosion in molten salt, showing the interlinked voids along the GB.

File Name: Supplementary Movie 2
Description: FIB-SEM 3D reconstruction on a larger volume in representative Ni-20Cr sample after corrosion in molten salt showing the gigantic wormhole network along the GB.

File Name: Supplementary Movie 3
Description: Animation showing the DIGM process.

File Name: Supplementary Movie 4
Description: Monte Carlo simulation to visualize the dual role of DIGM on the salt penetration efficiency.

File Name: Supplementary Movie 5
Description: FIB-SEM 3D reconstruction of a representative 316L stainless steel (SS) sample after corrosion in molten salt showing the interlinked 1D penetrating voids along the GB.

File Name: Supplementary Movie 6
Description: FIB-SEM 3D reconstruction of a representative Incoloy 800H sample after corrosion in molten salt, showing the interlinked voids along the GB.

File Name: Supplementary Movie 7
Description: FIB-SEM 3D reconstruction of a representative CrMnFeCoNi high-entropy alloy (HEA) sample after oxidation, showing the interlinked 1D oxide along the GB.

File Name: Supplementary Movie 8
Description: TEM tomography reconstruction of a representative Ni-4Al sample after corrosion in Rhines pack condition, showing the interlinked 1D oxide along the GB.
